# Integrin Signaling, Cell Survival, and Anoikis: Distinctions, Differences, and Differentiation

**DOI:** 10.1155/2011/738137

**Published:** 2011-07-13

**Authors:** Pierre H. Vachon

**Affiliations:** ^1^Département d'Anatomie et de Biologie Cellulaire, Faculté de Médecine et des Sciences de la Santé, Université de Sherbrooke, Sherbrooke, QC, Canada J1H5N4; ^2^Thématique de Recherche en Physiopathologie Digestive du Centre de Recherches Cliniques Étienne-Le Bel, Centre Hospitalier Universitaire de Sherbrooke, Campus Fleurimont, Sherbrooke, QC, Canada J1H5N4

## Abstract

Cell survival and apoptosis implicate an increasing complexity of players and signaling pathways which regulate not only the decision-making process of surviving (or dying), but as well the execution of cell death proper. The same complex nature applies to anoikis, a form of caspase-dependent apoptosis that is largely regulated by integrin-mediated, cell-extracellular matrix interactions. Not surprisingly, the regulation of cell survival, apoptosis, and anoikis furthermore implicates additional mechanistic distinctions according to the specific tissue, cell type, and species. Incidentally, studies in recent years have unearthed yet another layer of complexity in the regulation of these cell processes, namely, the implication of cell differentiation state-specific mechanisms. Further analyses of such differentiation state-distinct mechanisms, either under normal or physiopathological contexts, should increase our understanding of diseases which implicate a deregulation of integrin function, cell survival, and anoikis.

## 1. Introduction

A complex assortment of regulatory systems is required for the embryogenesis and ontogeny, as well as for the maintenance, renewal, and repair, of the comprehensive array of organs and tissues which allow multicellular organisms like mammals to function and survive. One of such critical regulatory systems is programmed cell death (PCD) [1–6], best defined as being “*a sequence of events based on cellular metabolism that lead to cell destruction*” [1]. Such a laconic definition of PCD includes nevertheless three distinct processes of cell death: apoptosis (caspase-induced or caspase-independent), autophagic cell death (caspase-induced or caspase-independent), and programmed necrosis (caspase-induced or caspase-independent) [1, 2, 7–10]. The present paper will focus on specific aspects of the “caspase-induced apoptosis” side of PCD, particularly with regards to integrin-mediated signaling.

## 2. Cell Survival and Apoptosis: The Ying and Yang of Life and Death

Apoptosis is a finely tuned process that performs crucial roles in several embryogenetic and physiological processes, such as tissue development and homeostasis, as well as the removal of defective, damaged and/or obsolete cells [1–10]. It is now well understood that regardless of their state of being, normal cells are intrinsically wired by default to enter apoptosis. Consequently, apoptosis must be kept in a suppressed mode when not needed and, therefore, cells require survival signals in order to remain alive [1–12]. Depending on the cell type, the said signals will include those provided by specific growth factors and their receptors. Aside from the loss of survival signals, apoptosis can also be induced by selected proinflammatory cytokines, as well as through various insults such as those caused by free radicals, radiation, or DNA-damaging agents, among others [1–10]. In typical instances of apoptosis, the death throes of a dying cell comprise membrane blebbing, chromatin condensation, DNA degradation, organelle destruction, and cell shrinkage [1–10]. Ultimately, the process results in the formation of apoptotic bodies that are either phagocytosed (by macrophages or neighboring cells), or simply released into a lumen for subsequent evacuation [1, 2, 4, 7–9, 13].

**Figure 1:**
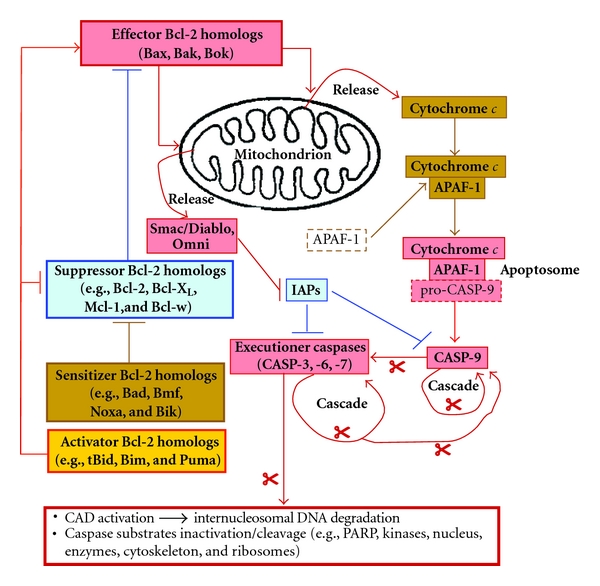
Intrinsic pathway of apoptosis. As decisional checkpoint of entry in apoptosis, Bcl-2 homologs perform functions—among many—in the integrity of the mitochondrion and thus regulating the formation of the apoptosome. Anti-apoptotic suppressor homologs inhibit their pro-apoptotic effector counterparts, preventing their translocation to the mitochondrion in order to create pores (and thus releasing cytochrome *c* and IAP inhibitors such as Smac/Diablo). Additional pro-apoptotic sensitizer and activator homologs act to inhibit the suppressors, although activators can furthermore interact with effectors to activate or enhance the functions of the latter. When the balance of Bcl-2 homologs is in favor of pro-apoptotics, effectors are free at the mitochondrion to homo-oligomerize, or hetero-oligomerize, with fellow effectors, and/or with activators, thus affecting the integrity of the mitochondrial membrane. APAF-1 and released cytochrome *c* can cooperate to dimerize pro-CASP-9, thus forming the apoptosome, resulting in massive CASP-9 activation and subsequent amplifying activation cascade of executioner caspases. Note that only the general outlines are shown here, for the sake of clarity. PARP, poly(ADP Ribose) polymerase; scissors: caspase-mediated cleavage.

The Bcl-2 (“B-cell chronic lymphocytic leukemia/lymphoma 2”) family of proteins—or homologs—is well known to constitute a critical decisional center of cell survival and apoptosis [6, 8, 11, 12, 14–17]. Some twenty members of this family have been identified so far in man [11, 12, 14–17]. While most Bcl-2 homologs are ubiquitously expressed regardless of the cell type [6, 8, 11, 12, 14–17], some may be prominently, or even selectively, expressed in a more restricted subset of tissues [6, 8, 11, 12, 14–17]. Alternately, some homologs will become expressed following specific apoptotic stimuli. A classic example of this is the p53-driven induction of the expression of the pro-apoptotics Puma and Noxa following DNA damage, consequently forcing cells to undergo apoptosis [6, 8, 11, 12, 14–17]. Bcl-2 homologs act primarily as apoptotic suppressors (“anti-apoptotics”; e.g., Bcl-2, Bcl-X_L_, Mcl-1), effectors (“pro-apoptotics”; namely, Bax, Bak, and Bok), activators (pro-apoptotics; e.g., Bid/tBid, Bim, Puma), or sensitizers (pro-apoptotics; e.g., Bad, Bmf, Bik, Noxa) (Figure 1) [6, 8, 11, 12, 14–17]. In general, the suppressors interact with the effectors in the cytosol to prevent effector translocation to mitochondria. Alternately, suppressors will bind effectors already present at mitochondrial membranes in order to prevent them from oligomerizing (Figure 1) [6, 8, 11, 12, 14–17]. Upon a given apoptotic stimulus, the balance of anti- and pro-apoptotic homolog expression and activities will be affected so that the sensitizers and activators gain the upper hand, therefore allowing both groups to inhibit suppressors (Figure 1) [6, 8, 11, 12, 14–17]. Additionally, activators will synergize with effectors to either help the latter translocate to mitochondria, or oligomerize with them at mitochondrial membranes (Figure 1) [6, 8, 11, 12, 14–17]. The end result is that effectors are now in force at mitochondrial membranes and are free to homo-oligomerize, or hetero-oligomerize, with activators or other effectors, in order to form pores (Figure 1) [6, 8, 11, 12, 14–17]. The consequent loss of mitochondrial membrane integrity leads to, among many things, the release of cytochrome *c* into the cytosol. Cytochrome *c* acts as a cofactor with APAF-1 (“Apoptosis Protease Activating Factor-1”) in recruiting the precursor/inactive form of the initiator caspase CASP-9, thus forming the apoptosome and consequently resulting in the activation of CASP-9 (Figure 1) [6, 11, 12, 14–18]. In turn, CASP-9 initiates an irreversible activation-amplification cascade of executioner caspases, such as CASP-3 and CASP-7, which will cleave their many substrates (e.g., actin, kinases, lamins, and keratins). Moreover, executioner caspases will activate by cleavage other apoptotic executioners such as CAD (“Caspase-Activated Dnase”), which enacts internucleosomal DNA degradation (Figure 1) [11, 12, 14–17, 19]. Lastly, numerous additional molecules that are implicated in the decision (or execution) of apoptosis have been identified to date, including cytosolic IAPs (“Inhibitors of Apoptosis”) which can inhibit caspases, as well as IAP inhibitors (e.g., Smac/Diablo, Omni) which are released during the loss of mitochondrial membrane integrity (Figure 1) [6, 8, 11, 12, 14–18, 20–23].

Thus, the fate of a cell depends principally on a tightly modulated balancing act between the anti- and pro-apoptotic activities from multiple Bcl-2 homologs [6, 8, 11, 12, 14–17]. Such a balance is established at its base through a modulation of the expression of various homologs according to the specific survival stimulus, cell type, and species concerned. However, post-transcriptional and/or post-translational modulations (e.g., alternative splicing, phosphorylation, sequestration) also contribute significantly to this balance [11, 12, 14–17, 24–26]. For instance, the phosphorylation of Bad on either of the S112, S136, or S155 residues inactivates its sensitizer functions, whereas phosphorylation of at least two of these residues furthermore leads to its degradation by proteasome. Another example is the case of Bid, which is normally expressed as an inactive precursor, and which consequently requires cleavage into its tBid form in order to enact its activator functions. Also of note are Bim and Bmf, two homologs that are typically sequestered in microtubules and actin microfilaments, respectively, therefore requiring their liberation from such captivity in order to perform their pro-apoptotic functions [11, 12, 14–17, 24–28].

It is of note that the establishment of the apoptosome (and consequent CASP-9 activation) constitutes what is generally recognized as the *intrinsic*, or “common,” pathway of apoptosis. This mode is typically induced by pro-apoptotic stimuli such as the loss of survival signals or critical failures in the operation of cellular processes (e.g., high-error DNA replication, plasma membrane peroxidation, DNA damage, protein misfolding, mitochondrial dysfunction) [1–12, 17, 18]. However, there is another mode of induction of apoptosis known as the *extrinsic* (or “death receptor,” “receptor-mediated”) pathway. This mode is induced by one type of pro-apoptotic stimulus, namely, the binding of “death ligands” (e.g., tumor necrosis factor (TNF)-*α*, FasL, or TNF-related apoptosis-inducing ligand/TRAIL) to specific “death receptors.” Once activated, these receptors lead to the recruitment of adaptor proteins such as FADD (“Fas-associated Death Domain protein”), which in turn recruit the precursor/inactive form of the initiator caspase CASP-8 in order to form the DISC (“Death Inducing Signaling Complex”). Consequently, this results in the activation of CASP-8 [10–12, 17, 29–32]. In cells with a high density of death receptors and/or high expression of pro-CASP-8, the DISC-induced activation of CASP-8 will be of sufficient intensity to bring about a subsequent amplifying activation cascade of executioner caspases, therefore rendering the process irreversible. However, in cells with a low density of death receptors and/or low expression of pro-CASP-8, the DISC-induced activation of CASP-8 will be instead of low intensity, in turn leading to low-intensity executioner caspase activation. In this situation, CASP-8 (and/or executioner caspases) will then have to (a) cleave Bid into its activator-functional tBid form, (b) destabilize actin microfilaments and microtubules by cleavage to allow the release of Bmf and Bim, and/or (c) cleave microtubule-sequestered Bim in order to directly release it. These altogether lead to a subsequent shift in the balance of anti- and pro-apoptotic Bcl-2 homolog activities, in effect causing a translocation of the death signal to mitochondria for the formation of the apoptosome and activation of CASP-9—and thus only then rendering the process irreversible [10–12, 14–17, 29–32]. The relevance of these considerations will become apparent in the following sections.

## 3. Integrins and Cell Survival: Grasping Desperately for Dear Life

Similarly to growth factors and their receptors, cell-extracellular matrix (ECM) interactions play a major role in regulating the various known cellular processes, including the maintenance of cell survival [12, 33–38]. The biological functions attributed to cell-ECM interactions are mediated primarily by integrin type transmembrane receptors [12, 33–39]. So far, 18*α* subunits and 8*β* subunits have been identified in humans, with *α* subunits noncovalently associating with *β* subunits in order to form 24 distinct heterodimeric (*αβ*) receptors with differing ligand specificities [39–44]. Some *α* and *β* subunits can undergo post-transcriptional alternative splicing, or post-translational proteolytic processing, resulting largely in variants with alterations in their cytoplasmic tails in order to add further versatility to their roles and functions [39–43].

Integrins can be divided into three main functional groups: cell-cell adhesion integrins, vascular integrins, and cell-ECM adhesion integrins. It is those integrins that have the *β*1 subunit in common which constitute the majority of receptors for ECM components [39–43]. Also of this group is the *α*6*β*4 integrin, which is expressed exclusively in epithelial cells [39, 44]. The repertoire of integrins (and variants) expressed by a given cell is dependent on the contexts of ECM composition, tissue-type, and species concerned [33–44]. Taking into account that some ECM components may be bound by more than one integrin, it is therefore not surprising that integrin-mediated cellular responses to the ECM are quite varied and multifaceted [39–44]. To this effect, the initial observations that a cell's anchorage to its ECM constitutes a critical factor for its survival [45, 46] were quickly followed by the realization that distinct ECM components and integrins are selectively implicated in the promotion of cell survival, depending on the tissue and species studied [33–35, 39, 44, 47–49]. For instance, laminin-211 promotes the survival of human and mouse skeletal myocytes via the *α*7B*β*1D integrin (a laminin receptor), whereas laminin-511 and fibronectin fail to support myocyte survival [50–53]. Also in myocytes, laminin-211 upregulates the expression of Bcl-2 and Bcl-X_L_, promotes the S112 phosphorylation of Bad, and downregulates the expression of Bax and Bak, whereas laminin-511 enacts the exact opposite effects [53]. Additionally, laminin-111 can actually replace laminin-211 to promote the survival of mouse skeletal myocytes [54–57], but not human ones [50], and does so at least in part via the *α*7B*β*1D integrin [54, 57]. 

The binding of an integrin to its ECM ligand produces not only a physical link with the cytoskeleton, but also generates a vast range of transduction signals which affect cell behavior, cell shape, and gene expression [12, 33, 34, 36, 43, 47–49, 58–61]. It is now well established that a given repertoire of expressed integrins not only engenders distinct signals for a specific cell type, but also exerts a differential modulation of cellular processes within the same tissue [33–44, 47, 48, 58–61]. Although well advanced, our understanding of the exact molecular bases of integrin-mediated signaling remains incomplete [58–61]. It is nevertheless known that an increasing number of pathways such as the phosphatidylinositol 3-kinase (PI3-K)/Akt pathway, the MEK (“Mitogen-Activated Protein Kinase (MAP) Kinase”)/Erk (“Extracellular Regulated Kinase”) pathway, or the SAPKs (“Stress-Activated MAP Kinases”) JNK (“c-Jun N-terminal Kinases”) and p38, can be activated alone or in combination according to the repertoire of integrins expressed by a given cell [12, 34, 36, 43, 48, 58–62].

**Figure 2:**
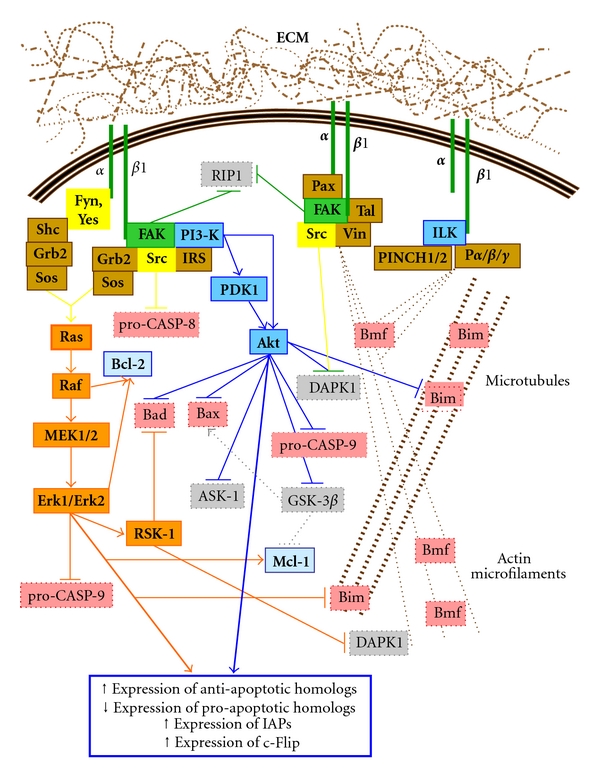
*β*1/Fak/Src integrin signaling, cell survival, and suppression of anoikis. Binding of an (*α*)*β*1 integrin to its ECM ligand primarily allows the recruitment and activation of Fak. On the one hand, Fak activates PI3-K directly or indirectly via Src and/or an IRS, leading to activation of Akt. Activated Akt then performs various cell survival functions. On the other hand, Fak may directly recruit Grb2-Sos, or indirectly via Src, leading to the activation of the small GTPase Ras and thereby stimulating the Raf/MEK/Erk pathway. This latter pathway can also be stimulated by certain partner *α* subunits of *β*1, through the recruitment/activation of Fyn or Yes, two other Src family members, and their subsequent recruitment of p52^Shc^-Grb2-Sos. The Raf/MEK/Erk pathway then likewise performs cell survival functions. Additionally, the stimulation of the PI3-K/Akt and/or Raf/MEK/Erk pathways generally results in an upregulation of Bcl-2 expression for the anti-apoptotic homologs and a down-regulation for the pro-apoptotic homologs, as well as an upregulation of IAP expression and other apoptosis suppressor molecules, such as c-Flip. Alternatively, Fak and/or Src will participate in the recruitment of paxillin, talin, vinculin to assemble focal adhesions in direct association with actin microfilaments. Similarly, ILK will be engaged by *β*1 integrins to assemble and regulate focal adhesions along with its PINCH and parvin partners, whereas ILK can also contribute in microtubule assembly/stability. The overall stabilization of the cytoskeleton enables the sequestration of the pro-apoptotic homologs Bmf and Bim. Note that various other survival signaling pathways stimulated by integrin *β*1/Fak/Src signaling (e.g., p130^CAS^-Nck-PAK, and PKC) are not shown here for reasons of clarity and conciseness. P*α*/*β*/*γ*, parvin *α*, *β*, *γ*; Pax, paxillin; PINCH1/2, particularly interesting new cysteine-histidine rich protein-1/-2; Tal, talin; Vin, vinculin.

However, unlike most growth factor receptors, integrins are nonkinase receptors. Consequently, integrins require kinase proxies to enact signal transduction following their activation. To this effect, signaling by *β*1 integrins owes largely to the recruitment and activation of Fak (“Focal Adhesion Kinase”) at the cytoplasmic tail of the *β*1 subunit. In turn, Fak typically recruits and activates Src [12, 33, 34, 36, 48, 58–73]. Conversely, Src may be the first to be recruited and activated at the cytoplasmic tails of *β* subunits [62, 71, 74] and in turn recruit/activate Fak [62, 63, 65, 67, 71]. Such integrin-mediated Fak/Src signaling allows for the downstream engagement of a plethora of pathways, largely due to the formation of diverse signaling cassettes through the recruitment by Fak and/or Src of an increasing array of signaling molecules. These can include adaptors (e.g., Sch, Grb2), scaffolders (e.g., IRS-1—“Insulin Receptor Substrate-1”), nucleotide exchangers (e.g., SoS), small GTPases (e.g., Ras), and other kinases (e.g., Cas) (Figure 2) [62–73, 75]. Similarly, integrin-mediated Fak/Src signaling contributes greatly in the assembly of focal adhesions via their interactions with a mounting number of partners (e.g., paxillin, talin, and vinculin), in order to not only create a bridge between the ECM and the actin cytoskeleton, but also to regulate the stability and organization of actin microfilaments [48, 58–65, 69, 71, 75, 76]. This enables, or maintains, the sequestration of Bmf and/or Bim (Figure 2). The modulation of integrin-mediated signaling and focal adhesion assembly can also be generated via certain *α* subunits (e.g., activation of Src family members Fyn or Yes), via the association with membrane proteins (e.g., caveolin), or via other adaptors and kinases, such as ILK (“Integrin-Linked Kinase”) (Figure 2) [34, 39, 43, 48, 58–62, 76–78]. Incidentally, the epithelial *α*6*β*4 integrin likewise participates in the stimulation of pathways such as PI3-K/Akt and/or MEK/Erk via its engagement of Src (but not Fak) and furthermore is a chief contributor in the formation of hemidesmosomes—anchoring complexes which link physically the ECM to keratin intermediate filaments, thus further organizing/stabilizing the cytoskeleton of a cell [39, 44, 58, 79, 80]. 

Overall, signal transduction by integrins differs little from that of growth factor receptors with tyrosine kinase activity (“RTKs”). It is therefore not surprising that there is much chatter between RTK- and integrin-mediated signaling, the two often cooperating in the regulation of various cell processes such as proliferation, migration, and survival [12, 33, 34, 39, 43, 44, 58–73, 77, 79–87]. To this effect, integrins can enact “inside-out” activation of RTK signaling largely through their own engagement of Fak, Src and/or ILK [62–73, 77–79, 82–87]. Reciprocity being the rule, RTKs can perform inside-out activation of integrin signaling, mainly through their own engagement of Src [64–66, 69–71, 86, 87]. Therefore, the already wide range of integrin-mediated signals can be expanded vastly further through such cross-talk with various growth factor receptors. 

The precise molecular mechanisms governing the promotion of cell survival by integrins remain to be fully elucidated. Nonetheless, PI3-K/Akt and Ras/Raf/MEK/Erk constitute the best known cell survival-promoting pathways among the assorted ones identified so far as being engaged by integrin-mediated Fak/Src signaling [12, 33–36, 47, 58–66, 68–70, 72, 73, 80, 81, 88–101]. With regards to the PI3-K/Akt pathway, Akt is well known for its numerous cell survival functions such as the inhibitory phosphorylation of Bax (on the S184 residue), Bad (on S136 preferentially, but on S112 as well), and Bim (on S87), as well as the phosphorylation of pro-CASP-9 (on S196) to suppress its activation and the inhibitory phosphorylation of GSK-3*β* (“Glycogen Synthase Kinase-3*β*”; on S9) which, when active, positively phosphorylates Bax (on S163) and negatively phosphorylates Mcl-1 (on S155, S159 and T163) (Figure 2) [11, 12, 14–17, 24–26, 34, 81, 88–94, 101]. Although ILK contributes in the formation of integrin-mediated focal adhesions by binding directly to the cytoplasmic tail of the *β* subunits of activated receptors [76–78], it has also been shown to be PI3-K-dependent for its activation and capable of acting as an upstream contributor to the activation of Akt [82–84, 88, 89, 91]. However, with the recent evidence that ILK is a pseudokinase [102, 103], its previously tacit kinase activation upon binding the cytoplasmic domain of integrin *β* subunits, as well as its kinase signaling roles in promoting integrin-mediated cell survival, remain therefore contentious [82–84, 102, 103]. At the very least, the scaffolding functions of ILK in focal adhesion assembly and actin microfilament linkage [76–78], as well as its implication in microtubule assembly [104], are likely to contribute in the sequestration of Bmf and Bim (Figure 2). Similarly, the said scaffolding functions of ILK can be used by integrins to cross-talk with RTKs, thus allowing for its contribution in RTK/integrin cooperative signaling in promoting cell survival [77, 78, 82–86].

Regarding the Ras/Raf/MEK/Erk pathway, Raf can phosphorylate Bcl-2 (on T69/S70) in order to potentialize its suppressor functions, whereas RSK-1 (“p90 Ribosomal S6 Kinase-1”), a usual effector of Erk1/Erk2, enacts an inhibitory phosphorylation of Bad (on S112) (Figure 2) [11, 12, 14–17, 24–26, 81, 95–99, 101, 105–107]. Additionally, Erk1/Erk2 themselves may phosphorylate Bcl-2 (presumably on T69/S70) to potentialize its suppressor functions, Mcl-1 (presumably on S159/T163) to protect it from degradation, Bim (on S69 and/or S87) to inhibit its activator functions and pro-CASP-9 (on T125) to prevent its activation (Figure 2) [11, 12, 14–17, 24–26, 81, 95–99, 101, 105, 106]. The PI3-K/Akt and Ras/Raf/MEK/Erk pathways can furthermore cross-talk with each other in order to cooperatively promote cell survival. For instance, Ras can activate PI3-K, which in turn results in the activation of Akt. Moreover, PDK1 (“Phosphoinositide-Dependent Kinase-1”), another effector of PI3-K, can activate RSK-1 [88, 94, 106, 107]. In any event, the engagement of PI3-K/Akt and/or Ras/Raf/MEK/Erk results generally in an up-regulation of the expression of anti-apoptotic Bcl-2 homologs and IAPs, as well as a down-regulation of the expression of pro-apoptotic Bcl-2 homologs (Figure 2) [11, 12, 14–17, 21, 22, 60, 81, 88–99]. Additionally, the expression of c-Flip (“cellular FADD-like IL-1*β*-converting enzyme-inhibitory protein”), which blocks the formation of the DISC by binding to FADD [12, 30, 32, 81], is likewise up-regulated [12, 60, 81, 88–99].

Hence, the importance of Fak and/or Src in the integrin-mediated promotion of cell survival is intimately linked with their pivotal role in the engagement of pathways such as PI3-K/Akt and/or Ras/Raf/MEK/Erk (Figure 2), depending on the composition of the signaling cassettes they help engender and the specific integrin receptors implicated, as well as according to the cell type and species studied [12, 34, 43, 47, 48, 58–73, 80]. Interestingly, Fak and Src can also contribute directly in the promotion of cell survival, aside from their usual roles in the integrin-mediated assembly of focal adhesions and engagement of survival-promoting pathways (Figure 2). Indeed, Src can phosphorylate pro-CASP-8 (on Y380) to suppress its activation [108], whereas Fak can bind RIP1 (“Receptor-Interacting Protein-1”; a.k.a. RIPK1) in order to prevent its recruitment of FADD and consequent formation of the DISC [109, 110]. All in all, it is therefore more than ever undisputable that a cell's integrin-mediated anchorage to its ECM constitutes a powerful, multilayered, and complex device whose most significant purpose is to ensure survival. It should be noted here that the already complicated nature of the regulation of cell processes is strikingly enhanced by the implication of specific kinase isoforms and/or family kinase members, therefore often resulting in distinct—or even differing—roles by the pathways in which they participate. PI3-K isoforms complexes (4 catalytic subunit isoforms, 3 regulatory subunit isoforms), Akt isoforms (−1 to −3), RSK isoforms (−1 to −4), IRS isoforms (−1 to −4), or Shc (p46^Shc^, p52^Shc^, p66^Shc^) and Raf (A- to C-) family members, constitute but a few examples identified to date in humans [71, 94–96, 98, 101, 105–107, 111–113]. That these can be selectively expressed depending on the tissue and species, in addition to the fact that they can perform specific functions even within the same given cell type, further emphasizes the reality of the vast intricacies that underlie the regulation of cell processes—including of course cell survival, whether integrin mediated or otherwise.

## 4. Integrins and Anoikis: Making Sure That Letting Go Really Means the End

Considering the importance of integrin-mediated cell-ECM interactions in driving cell survival, it is therefore not surprising that the disruption, or loss, of integrin binding induces PCD—namely, a form of caspase-dependent apoptosis that is termed *anoikis* (a.k.a. “detachment-induced apoptosis” or “integrin-mediated death”) [10, 12, 33–36, 46, 47, 49, 60, 81, 101, 108, 114–120]. Like apoptosis and PCD in general, anoikis performs important roles during organogenesis, as well as in tissue maintenance and renewal [33–35, 38, 49, 79–81, 101, 117–122]. For example, the involution of mammary glands and the renewal of the epidermis, as well as that of the intestinal epithelium, implicate the induction of anoikis in obsolete cells [101, 121–126]. In this respect, it is now recognized that cells are endowed with an innate/default anchorage-dependent surveillance system meant to ensure that all integrins expressed by them do interact with their respective ECM ligands, thus inducing anoikis when that is not the case. In other words, any cell that strays accidentally or otherwise from its assigned position within its given tissue, either by interacting with a “wrong” ECM or by losing anchorage to its own ECM, is targeted for death [12, 36, 60, 108, 115, 117].

**Figure 3:**
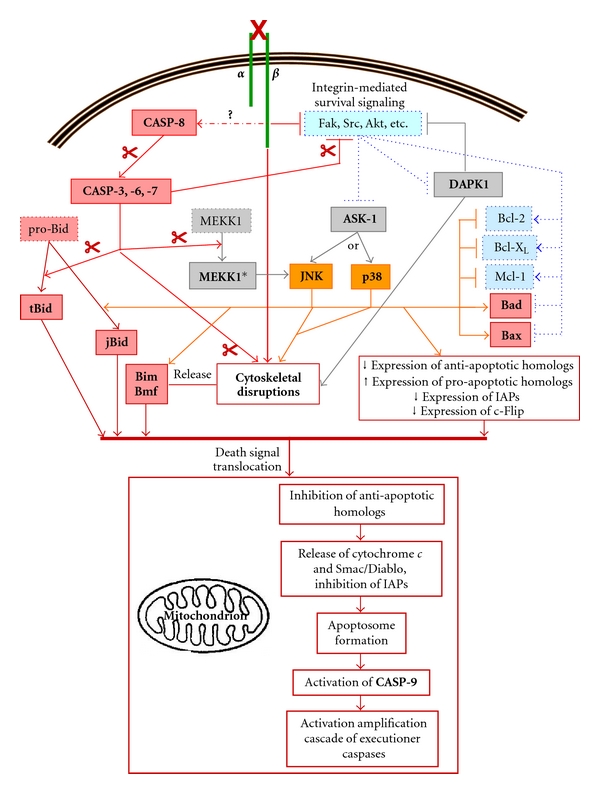
Common pathway of anoikis induction. The loss of integrin-mediated adhesion results in the faltering of survival signals (Fak, Src, Akt, etc.), as well as in the activation of the initiator caspase CASP-8 through a mechanism which remains unclear (“?”). This in turn leads to a “low-intensity” activation of executioner caspases. On the one hand, these caspases will activate the pro-apoptotic homolog Bid and further inactivate pro-survival kinases associated with integrin signaling, among other things. On the other hand, the resulting cytoskeletal disruptions from the loss of integrin binding, further exacerbated by excutioner caspases, will release Bim and Bmf. Moreover, SAPKs (JNK and/or p38 isoforms) and DAPK1 are activated, presumably furthering cytoskeletal disruption, among other apoptotic functions. Overall, these events contribute to the translocation of the death signal to mitochondria, resulting ultimately in the formation of the apoptosome (see Figure 1) and thus rendering the process irreversible. Note that only the general outlines are shown herein, for the sake of concision. MEKK1*, constitutive active MEKK1 following caspase-cleavage; scissors: caspase-mediated cleavage.

Anoikis constitutes overall a “four-punch hit” against cell survival that implicates elements from both the intrinsic and extrinsic pathways of caspase-dependent apoptosis. The first “punch” comes from the deactivation of Fak and/or Src, leading to a disengagement of pathways such as PI3-K/Akt and Ras/Raf/Mek/Erk (Figure 3). Hence, the numerous pro-survival roles performed by these pathways (Figure 2) undergo failure. The second “punch” comes from the concomitant disassembly of anchoring focal adhesions (and/or hemidesmosomes), in large part by the loss of integrin-mediated engagement of Fak, Src, and/or ILK (Figure 2), thus greatly destabilizing the cytoskeleton and consequently allowing the release of Bmf and Bim (Figure 3). Additionally, the presence of p66^Shc^ (a member of the Shc family of adaptor proteins [113]) at focal adhesions can induce an elevated/sustained activation of the small GTPase RhoA, which in turn contributes likewise in the destabilization of the cytoskeleton through effectors that remain to be fully identified [127]. Interestingly, such “anoikis-sensitizing” function of p66^Shc^ appears to be independent of its better known ability to translocate to mitochondria that is typically part of its apoptosis-inducing roles [113, 127].

The third “punch” consists in the activation of “apoptotic” kinases [12, 36, 81, 101, 108, 115, 117–120, 128]. The roles of the SAPKs JNK and p38 in anoikis remain somewhat ambiguous [95, 96, 98, 101, 117, 129–134]. Three isoforms for JNK (−1 to −3) and four for p38 (*α*, *β*, *γ*, and *δ*) have been identified to date, all of which can be selectively expressed depending on the cell type and species studied [95, 96, 98, 129–134]. Accordingly, JNK and p38 will contribute to cell survival or apoptosis/anoikis, or neither, according to cell type as well as in an isoform-selective manner [95, 96, 98, 129–134]. In addition, and again depending on the cell context as well as the specific stimulus, it will be either a JNK isoform, a p38 isoform, or an isoform of each, which will be implicated in cell survival or apoptosis/anoikis [95, 96, 98, 101, 129–136]. As example, JNK1 drives anoikis in canine kidney epithelial cells [128, 137, 138], but not JNK2 [128, 139]. Similarly, p38*α* is required for apoptosis/anoikis in rat cardiomyocytes [140] and mouse skeletal myocytes [52], whereas p38*β* contributes in the promotion of survival in the former [140], while playing no role to this effect in the latter [52]. Consequently, much remains to be understood of the implication of JNK and/or p38 in anoikis. It is however generally accepted that a prolonged/sustained activation of specific JNK and/or p38 isoforms can allow them more than enough time to enact their deadlier functions, therefore driving the cell death process [95, 96, 98, 101, 115, 118, 128–136]. To this effect, it is now well established that an elevated and/or sustained activation of an apoptotic JNK isoform, or p38 one, will result largely from the upstream activation of ASK-1 (“Apoptosis Signal-regulating Kinase-1”) (Figure 3) [95, 96, 98, 101, 115, 118, 129–136, 141, 142]. Incidentally, Akt phosphorylates ASK-1 (on S83) as part of its pro-survival functions, thus keeping the latter's activation in check (Figures 2-3) [88–93, 141, 142]. Little is known of the precise apoptotic roles, if any, that are enacted by each specific JNK and p38 isoforms [95, 96, 98, 129–136]. Nevertheless, it is recognized that apoptosis-induced JNK or p38 can generally perform numerous apoptotic-driving functions such as inducing/up-regulating the expression of FasL (death ligand of Fas) for autocrine “death stimulation,” contributing in the destabilization of cytoskeletal elements (such as microtubules and microfilaments) to induce/enhance the liberation of Bim and Bmf, contributing to the membrane blebbing that is characteristic of apoptosis, down-regulating the expression of IAPs and anti-apoptotic Bcl-2 homologs while up-regulating that of pro-apoptotic ones, and phosphorylating homologs either in the cytosol or at mitochondria (Figure 3) [12, 36, 81, 95, 96, 98, 101, 108, 114, 115, 117–120, 129–136]. Apoptotic phosphorylating functions have been mostly characterized so far in the case of JNK (albeit not for any particular isoform), namely, the negative phosphorylation of Bcl-2 (on T69/S70/S87), Bcl-X_L_ (on S62 and/or T47/T115), and Mcl-1 (on S121/T163), the phosphorylation of Bax (T167) for its activation and mitochondrial translocation, the positive/potentiating phosphorylation of Bad (S128), the phosphorylation of Bid to produce an active jBid form (instead of the usual tBid one), the phosphorylation of Bim (on T56 and either S44 or S58) to prevent its sequestration or cause its release from microtubules, and the phosphorylation of Bmf (S58 or S77) to prevent its sequestration, or cause its release from microfilaments (Figure 3) [11, 12, 14–17, 24–28, 128–136, 141, 142]. Although similar apoptotic phosphorylations of Bcl-2 homologs have been reported for p38, the specific residues targeted are yet to be firmly identified (Figure 3) [11, 12, 14–17, 24–28, 129–136, 141, 142].

Another apoptotic kinase family of note is the DAPK family (“Death Associated Protein Kinase”; especially DAPK1) [143–146]. Although the exact mode of activation for DAPK1 remains poorly understood, it has been observed that death receptor signaling (namely, the receptors for TNF-*α* and FasL) can lead to DAPK1 activation [143–148]. Incidentally, Src, Akt, and RSK-1 may phosphorylate DAPK1 (on Y491/Y492, S308, and S289, resp.) to maintain it in a repressed, inactive state (Figure 2) [143–148]. Accordingly, the activation of integrins and Fak results in the suppression of DAPK1 activation [149]. The apoptotic functions of DAPK1 likewise remain to be fully elucidated. It is however known that activated DAPK1 contributes greatly to the destabilization of the cytoskeleton and is critical for membrane blebbing (Figure 3) [143–146]. DAPK1 may furthermore deactivate integrins through an inside-out mechanism that involves the displacement and replacement of talin at the cytoplasmic tail of *β* subunits, consequently resulting in disassembly of focal adhesions (Figure 3) [149–151]. Interestingly, DAPK1 phosphorylates Beclin-1, an autophagic cell death-driving factor [1–3, 7–9], in order to free it from inhibitory binding by Bcl-2 and Bcl-X_L_ [145, 146]. Considering that experimental attempts at inhibiting intrinsic pathway apoptotic effectors following the induction of anoikis often fail to protect cells, resulting instead in their Beclin-1-mediated autophagic death [2, 7–10, 118–120], the detachment-induced activation of DAPK1 is therefore likely to constitute a functional bridge between anoikis and autophagic cell death.

The fourth “punch” delivered against cell survival by the loss of integrin binding consists in the induction of the extrinsic pathway of apoptosis—that is, CASP-8 activation (Figure 3) [12, 36, 81, 101, 108, 115, 117–120]. Although the activation of CASP-8 constitutes an early/immediate event following the loss of integrin-mediated cell adhesion, the precise mechanisms that are responsible for such activation remain poorly understood. A part of the puzzle lies with previous observations that both pro-CASP-8 and activated CASP-8 are associated with the cytoplasmic tails of *β*1 and/or *β*3 subunits of unligated integrins [152, 153]. Interestingly, pro-CASP-8 is found already associated with the cytoplasmic tails of integrin *β* subunits under healthy/adhering conditions [108, 153], most likely due to the fact that pro-CASP-8 often complexes with Src following its inhibitory phosphorylation by the latter [108]. Nonetheless, the formation of a DISC at unligated integrins as the causal agent for CASP-8 activation remains contentious, despite reports from different cell types that CASP-8 activation can be FADD-dependent in anoikis [109, 110, 154–160] and that c-Flip can inhibit the process [156, 160, 161]. Indeed, with Fak and Src being downactivated following detachment from the ECM, RIP1 is freed from Fak [109, 110], and pro-CASP-8 is no more negatively phosphorylated by Src (see previous section) [108, 162]. Additionally, the concomitant downactivation of the PI3-K/Akt and/or Ras/Raf/MEK/Erk pathways leads to a down-regulation of c-Flip expression (see previous section and Figure 3) [12, 60, 81, 116–118, 120]. Thus, RIP1, FADD and pro-CASP-8 can complex to form the DISC [109]. However, it turns out that Fak-freed RIP1 forms a FADD-dependent DISC at the cytoplasmic domain of the death receptor Fas [110]. In hindsight, that is to be expected given that, in some cell types, the expression of FasL can be upregulated following the loss of integrin binding, or that anoikis can be attenuated/blocked by the inhibition of Fas [12, 81, 109, 110, 115, 116, 118–120, 154, 155, 160, 161]. Yet in the end, RIP1 does not form a DISC at unligated integrins [110]. Furthermore, it has been shown that FADD is not found associated with the cytoplasmic tails of *β*3 subunit-containing unligated integrins in human umbilical vein endothelial cells undergoing anoikis, despite the fact that pro-CASP-8 and activated CASP-8 are [152]. Most perplexing is what has been reported recently in human keratinocytes. In these cells, bound *β*1 integrins contain the *β*1A subunit variant, whereas the *β*1B variant is expressed in the cytosol and apparently not part of any functional heterodimeric receptor [153]. As can now be expected (see above), pro-CASP-8 is found associated with the cytoplasmic tails of *β*1A subunits when those cells are adhering [153]. Upon loss of ECM binding, *β*1A integrins are internalized so as to colocalize with the cytosolic *β*1B subunits, whereby pro-CASP-8 somehow shuttles from the tails of the former to those of the latter in order to undergo activation in a FADD-independent manner [153]. Hence, how exactly pro-CASP-8 and/or activated CASP-8 associate with *β* subunits of unligated integrins, as well as the precise mechanisms responsible for CASP-8 activation at these sites, remain open questions. Although it is not yet established whether DAPK1 can contribute to the formation of a DISC [143–148], the fact that activated DAPK1 binds to the cytoplasmic tail of *β* subunits of integrins it has deactivated [149–151] definitively raises suspicions to that effect, especially in light of the previous observations in keratinocytes. Then again, it may simply be that the mechanisms of CASP-8 activation following the induction of anoikis differ according to the combined contexts of the cell type, of the integrin repertoire expressed (including variants), and of the species concerned—not unlike everything else that has been discussed herein so far.

In any case, the activation of CASP-8 following the loss of integrin binding is typically of low-intensity. As a result, this leads to a likewise low intensity activation of executioner caspases (Section 2). Hence, anoikis implicates the necessary following events: (a) caspase-mediated cleavage of Bid into its active tBid form; (b) caspase-mediated cleavage of pro-survival kinases (e.g., Fak, Akt) for deactivation; (c) caspase-mediated liberation of Bmf and Bim (Figure 3) [12, 36, 81, 101, 108, 115, 117–120]. Moreover, MEKK1 (“Mitogen-activated protein kinase kinase kinase 1”), an upstream activator of the JNK pathway, may be cleaved by executioner caspases (namely, CASP-7) in order to generate a constitutive active form which is responsible for a consequent prolonged/sustained activation of JNK (JNK1 presumably, [128, 137, 138]), who then undertakes apoptotic phosphorylating functions (see above and Figure 3). Consequently, these events altogether effect a translocation of the death signal to mitochondria for the formation of the apoptosome and activation of CASP-9 (Figure 3). Interestingly, anoikis in normal cells is characterized by a delay in the irreversible commitment to the process following the triggering of its initial pro-apoptotic events [12, 46, 79, 81, 101, 114, 120, 128]. This explains why cells can be rescued from anoikis by reattachment within a limited time-span subsequent to their loss of anchorage [12, 46, 79, 81, 101, 114, 120, 128]. Such a “window of anoikis reversibility” varies in length depending on the cell type and species, thus ranging from some fifteen minutes to four hours [12, 46, 79, 81, 101, 114, 120, 128]. It is thought that a window of anoikis reversibility depends primarily on the time-span required for the death signal to reach mitochondria, according to the specific integrins (and variants) implicated, the precise determinants of cell survival/death in play (e.g., Bcl-2 homolog expression profiles, survival pathways/isoforms engaged, apoptotic kinases/isoforms involved, etc.), and the degree of cytoskeletal organization/cell polarity imparted not only by integrins (i.e., focal adhesions, hemidesmosomes), but as well by cell-cell interactions (e.g., E-cadherins). Indeed, the latter sensitize cells to anoikis by further linking/organizing the cytoskeleton, albeit contributing at the same time to cell survival signaling [12, 46, 81, 114, 115, 128, 163, 164]. Additionally, it has been observed that the activation of pro-survival kinases such as Src, Akt and/or Erk1/Erk2 undergoes a short (five to fifteen minutes in length), transient up-activation following the loss of anchorage. While the mechanisms responsible for these “deathly gasps” remain unclear, they are first and foremost considered to act as a protection against transient detachments from the ECM, such as those required during normal cell processes like migration or cytokinesis. Accordingly, these may also contribute in defining a window of anoikis reversibility [12, 46, 79, 81, 114, 120]. However, considering the recent evidence that an up-activation of MEK/Erk in normal cells can actually trigger the intrinsic apoptotic pathway [165, 166], or activate DAPK1 and a consequent autophagic cell death [148, 149, 165, 166], the question arises whether such “deathly gasps” may not in fact represent “poisoned fruits.” Be that as it may, it is clear that once CASP-9 activation occurs, any window of reversibility is shut and anoikis becomes irreversible—so much so, in fact, that attempts from this point on to inhibit the process result in Beclin-1-driven autophagic cell death (see above) [2, 7–10, 118–120]. To this effect, this may explain why the overexpression of Bcl-2 or Bcl-X_L_ alone can protect cells from anoikis [12, 36, 53, 81, 114, 117–120, 128, 137, 167], as such overexpression would not only manage to block the translocation of the death signal to mitochondria following detachment, but furthermore prevent the trigger of autophagic cell death by an overwhelming inhibitory binding to Beclin-1.

Given that normal cells require a sound anchorage to their ECM in order to live, and in view of the “four-punch hit” described above that occurs following the loss of integrin-mediated attachment, it is therefore usually considered that integrins suppress anoikis [12, 81, 101, 120–123]. This is well exemplified by the observations that the forced expression of dominant negative mutants of Fak and/or Src readily induces anoikis, whereas the forced expression of constitutive active mutants of either, or both, protects against it [12, 60, 63–73, 81, 99–101, 114, 119, 120, 168]. Likewise, similar results are obtained when mutants of kinases that are engaged by integrin/Fak/Src-mediated cell survival signaling are used, such as PI3-K or Akt [12, 60, 63–73, 81, 81, 88–99, 99, 100, 100, 101, 101, 114, 119, 120]. However, there is now evidence that some integrins may actually sensitize cells to anoikis. As example, the knockdown of the expression of *α*8*β*1 in human intestinal epithelial crypt cells results in the loss of vinculin at focal adhesions and confers a measure of anoikis resistance via an illicitly sustained activation of Fak [169]. Although it remains unclear by which mechanisms Fak activation is thus sustained, it is germane that vinculin has been shown to enforce the adhesion-dependent activation/deactivation of Fak [60, 67, 170, 171] and that the gene disruption of vinculin in F9 mouse embryonic carcinoma cells renders them resistant to anoikis, also via a sustained activation of Fak [170]. Another example of anoikis-sensitizing integrin is *α*v*β*3 in colon cancer cells, although it is not known how this integrin enacts such a sensitizing function [172, 173]. It is thought that anoikis-sensitizing integrins may be part of the above-mentioned anchorage-dependent surveillance system, as a means to further warrant the disposal of cells that detach from their ECM [169]. However, it is already clear that anoikis-sensitizing functions will be undertaken by specific integrins according to the cell type. Indeed, *α*v*β*3 is better known for its anoikis-suppressing roles in endothelial cells, among other cell types [174, 175], whereas *α*8*β*1 has already been reported to suppress anoikis in myofibroblasts [176].

Overall, the preceding considerations altogether demonstrate the multilayered complexities that underlie the regulation of cell survival, apoptosis, and anoikis. Moreover, these include further mechanistic distinctions according to the contexts of the composition of the ECM, the specific integrins implicated, the cell type, and the species. However, studies in recent years in one tissue in particular, the human intestinal epithelium, have unearthed yet another level of intricacy in the regulation of these processes, namely, the implication of distinct mechanisms according to the state of cell differentiation.

## 5. Differentiation State-Specific Regulation of Cell Survival, Anoikis: Difference Is the Key

**Figure 4:**
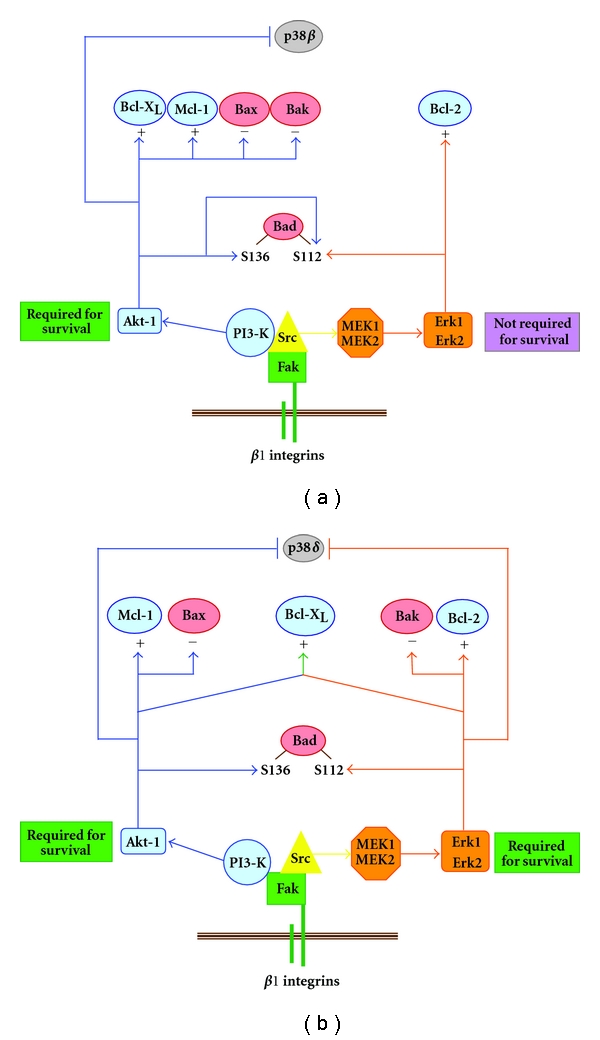
Differentiation state-specific mechanisms of integrin *β*1/Fak/Src-mediated control of human intestinal epithelial cell survival and anoikis. In undifferentiated enterocytes (a), *β*1 integrin/Fak signaling recruits Src which acts as a cornerstone in the subsequent engagement of PI3-K/Akt-1 and MEK/Erk in the suppression of anoikis. However, in contrast to PI3-K/Akt-1, MEK/Erk is not required for survival due to its non-to-marginal roles in the regulation of the expression/functions of Bcl-2 homologs and its noninvolvement in suppressing the activation of the enterocyte undifferentiated state-selective apoptotic kinase isoform p38*β*. In differentiated enterocytes (b), MEK/Erk remains Src dependent but not PI3-K/Akt-1 (which however remains Fak-dependent). PI3-K/Akt-1 and MEK/Erk are now both required for survival, as they both play major roles in the regulation of Bcl-2 homolog expression/functions as well as in suppressing the activation of the enterocyte differentiated state-selective apoptotic kinase isoform p38*δ*. +, up-regulation of expression; −, down-regulation of expression.

The intestinal epithelium is an elegant and valuable physiological system for understanding the functional connections between integrin-mediated cell-ECM interactions and the cell state [101, 125, 177–181]. The continuous renewal of this simple columnar epithelium occurs along a well-defined unit, the crypt-villus axis. This unit consists generally in two cell populations: the proliferative, immature cells of the crypt and the differentiated cells of the villus [125, 126]. As part of the dynamic process of intestinal epithelial renewal, obsolete enterocytes enter anoikis upon reaching the apex of the villi, as a means of exfoliation [101, 125, 126, 180]. For their part, crypt cells can occasionally undergo apoptosis in order to evacuate daughter cells that are damaged or defective [101, 125, 126, 180]. Such apparent contrast of destiny between undifferentiated and differentiated enterocytes along the crypt-villus axis, coupled with their specific profiles of expression of Bcl-2 homologs which are established during the differentiation process [101, 125, 182–186], initially introduced the concept of a distinct modulation of cell survival and apoptosis according to the state of differentiation [101, 125, 184]. This concept so far has been demonstrated mostly in human intestinal epithelial cells [101, 125]. For instance, the individual expression of Bcl-2 homologs is subjected to specific regulatory mechanisms depending on the differentiation status of enterocytes [167, 185–187]. As example, the PI3-K/Akt-1 and MEK/Erk pathways are selectively implicated in the promotion of enterocytic survival according to the state of differentiation, including with regards to their modulation of the expression/activity of Bcl-2 homologs (Figure 4) [167, 185–187]. In this respect, PI3-K/Akt-1, but not MEK/Erk, is critical for the survival of undifferentiated/crypt enterocytes, whereas both pathways are vital for the survival of differentiated/villus ones (Figure 4) [167, 185–187].

The obvious follow-up question is whether the concept of a distinct modulation of cell survival and apoptosis according to the state of differentiation applies as well to integrin-mediated cell survival and anoikis. In effect, human intestinal epithelial crypt and villus cells express differential profiles of integrins as they interact with specific basement membrane components, which are likewise deposited differentially along the crypt-villus axis [125, 177–181, 184, 188]. As expected when considering the normal fate of villus cells (see above), it turns out that differentiated/villus enterocytes are more susceptible to anoikis than their undifferentiated/crypt counterparts [185, 189, 190]. Although crypt cells express the *α*8*β*1 receptor as an anoikis-sensitizing integrin [169], the villus cells are nonetheless highly polarized in addition to bearing E-cadherin adherens junctions, in stark contrast to the former [163]. Moreover, such distinctions in anoikis susceptibility between crypt and villus enterocytes translate into differentiation state-specific mechanisms of integrin-mediated cell survival and anoikis (Figure 4) [167, 185, 186, 189–191]. For instance, *β*1 integrins, Fak, and Src distinctively modulate the expression/activity of Bcl-2 homologs depending on the enterocytic differentiation status (Figure 4) [167, 185, 186, 190]. Furthermore, *α*2*β*1, *α*5*β*1, and *α*6A*β*1 are required for the survival of undifferentiated enterocytes, whereas *α*3*β*1, *α*6B*β*1 and *α*6B*β*4A are required for the survival of differentiated ones [185, 186] (S. Thibodeau and P.H. Vachon, manuscript in preparation). Similarly, the engagement of PI3-K/Akt-1 is integrin *β*1/Fak/Src-dependent in undifferentiated cells, but Src-independent in differentiated ones, whereas the engagement of MEK/Erk remains integrin *β*1/Fak/Src-dependent regardless of the state of differentiation (Figure 4) [167, 185, 186, 189–191]. To this effect, the integrin *β*1/Fak/Src/PI3-K/Akt-1 pathway antagonizes the apoptotic activation of p38*β* in undifferentiated enterocytes, whereas the integrin *β*1/Fak/PI3-K/Akt-1 and integrin *β*1/Fak/Src/MEK/Erk pathways both contribute in antagonizing that of p38*δ*, in differentiated ones (Figure 4) [167, 186, 189].

Consequently, the regulation of integrin-mediated cell survival and anoikis is indeed subjected to differentiation-state-specific mechanisms (Figure 4). However, the overall concept of differentiation state-specific controls of cell survival, apoptosis, and anoikis does not constitute a peculiarity of the intestinal epithelium, since it evidently applies to other tissues—albeit not without obligatory cell type- and/or species-dependent distinctions. For instance, skeletal muscle myoblasts require fibronectin and *α*5*β*1A in order to survive, whereas myocytes require instead laminin-211 and *α*7B*β*1D [50–53]. Furthermore, myoblasts depend on an integrin-driven Fak/Src/MEK/Erk pathway for their survival, whereas myocytes depend instead on an integrin-driven Fyn/PI3-K/Akt-2 one [52, 53, 192, 193]. A fundamental challenge now presenting itself lies in the search for answers to three broad questions regarding such differentiation state-specific mechanisms of cell survival, apoptosis and anoikis, namely: (a) the “why” for the existence of such distinct mechanisms according to the state of cell differentiation; (b) the “how” of these distinct mechanisms (i.e., the further functional identification of specific extracellular ligands, integrins and molecules/pathways involved, as well as their respective differentiation-specific roles in the suppression or induction of apoptosis/anoikis); (c) the “in what capacity” these same differentiation-specific mechanisms contribute in the emergence of diseases when they become deregulated.

## 6. Integrins, Anoikis, and Disease: Too Much or Not Enough, It's Still Unhealthy

As in the case of apoptosis, there is an increasing number of pathological disorders that are characterized by a deregulation of integrin-mediated cell survival, and anoikis signaling [35, 39, 44, 49, 117, 118, 120, 194]. Certain forms of muscular dystrophy and epidermolysis bullosa constitute classic examples of pathologies that are caused primarily by a deregulated induction of anoikis [39, 44, 79, 80, 195–197]. Accordingly, the need to understand the regulation of cell survival and apoptosis has gained much importance in cancer research, especially since the acquisition of a resistance to anoikis constitutes a critical step in tumor progression—particularly in the emergence of invasive and metastatic cells [86, 116, 118–120, 174, 188, 198–200]. Incidentally, cancer cells are notorious for exhibiting major alterations in their repertoire of expressed integrins [49, 62, 80, 86, 174, 180, 188, 199, 200], as well as in their surrounding ECM [35, 49, 180, 188, 200]. Moreover, metastatic cells are infamous for displaying a marked resistance to anoikis [86, 116, 118–120, 174, 188, 198–200]. To this effect, much attention has been given to Fak and Src in tumor progression [63–73, 201], as well as to downstream pathways engaged by them, such as PI3-K/Akt and MEK/Erk [88–99]. Along with these, virtually every known player in the intrinsic and extrinsic pathways of apoptosis is likewise being scrutinized, in order to not only seek an eventual understanding of the acquisition of resistance to anoikis (and/or apoptosis) by cancer cells, but as well as to identify molecular targets that are susceptible to shut down such problematic resistance. Thus, anoikis-sensitizing integrins may be added to this expanding list, in view of the observations that anoikis-resistant colon cancer cells do not express *α*8*β*1, whereas forcing its expression reinstates in them a good measure of susceptibility to anoikis [169]. Similarly, colon cancer cells that are anoikis-sensitive acquire a resistance to the process following their loss of expression of *α*v*β*3 [172]. However, the cross-talk between integrins and RTKs further complicates matters greatly, since the deregulated activity of an RTK can confer resistance to anoikis by maintaining/enhancing integrin-mediated cell survival after the loss of attachment, via inside-out signaling [12, 34, 86, 87, 116, 117, 119, 120, 174, 175]. As one example among many, colon cancer cells that strongly express EGF/TGF-*α* (“Epidermal Growth Factor/Transforming Growth Factor-*α*”) display a resistance to anoikis by at least in part sustaining the activation of Src via autocrine stimulation of EGFR (“EGF Receptor”), therefore allowing Src to maintain functional interactions with Fak and consequently sustaining a Fak/Src-mediated suppression of anoikis [202]. In this respect, the deregulation of RTK signaling is well known to induce epithelial-mesenchymal transition (EMT) in normal epithelial cells, a phenotypic change that is considered a prerequisite for the acquisition of anchorage-independent growth and resistance to anoikis, consequently leading to the emergence of invasive and metastatic cancer cells [62, 66–73, 85–87, 116, 119, 120, 200, 201, 203].

Hence, the task of elucidating the mechanisms of resistance to anoikis in cancer cells, let alone identifying commonalities, is evidently proving to be a daunting one—and that is without taking into account the inherent molecular differences between the various types of cancers, as well as the cellular and molecular heterogeneity that is invariably found within any given cancer. Nevertheless, increasing our knowledge of those determinants which control cell survival, apoptosis, and anoikis within a specific tissue will, in turn, add further to our comprehension of apoptosis/anoikis-linked diseases of said tissue, in addition to paving the way toward the design of targeted molecular therapeutic approaches that are both tissue- and disease-selective. To this end, the inclusion of acquiring a better grasp of differentiation state-specific distinctions in the integrin-mediated regulation of cell survival and anoikis in such research endeavors would not only allow for a more complete understanding of a given tissue's normal physiology, but furthermore provide a full accounting of the physiopathological underpinnings of its apoptosis/anoikis-linked diseases—including tumorigenesis and tumorigenic progression. Incidentally, cancerous intestinal epithelial cells display integrin/Fak/Src anoikis-suppressing signaling features that are similar to either their normal undifferentiated or differentiated counterparts, and do so regardless of their own degree of cell dedifferentiation [202]. Whether or not cancer cells of other tissues do likewise remains, however, to be confirmed.

## 7. Post-Mortem and Conclusions

Cells require survival signals in order to live, and interactions with their ECM through integrins constitute a critical contributor to this effect. Integrin-mediated cell survival and anoikis comprise a multifaceted and multilayered surveillance mechanism which is responsible for upholding the correct position of cells within their respective tissues, thereby sentencing to death any cell that would stray from its assigned position by either interacting with an inappropriately composed ECM or by losing anchorage to its own ECM. Thus, anoikis partakes greatly in the configuration and preservation of the proper functional organization of tissues, thereby ensuring the disposal of cells that assume a roguish behavior with regards to their precise integrin-ECM adhesion requirements for survival—whether this occurs accidentally in the course of normal physiological processes, such as tissue renewal, or otherwise.

It is undeniable that the regulation of cell survival and anoikis implicates distinct mechanisms according to the composition of the ECM, the repertoire of integrins (and variants) expressed, the degree of cell polarity (i.e., cytoskeletal organization) conferred at least in part by specific ECM-integrin interactions, the transduction pathways engaged and/or suppressed through signaling by individual integrins, the isoforms and/or family members participating in such pathways, the RTKs expressed which may, or may not, interfere with their intrusive cross-talk, and the various regulators/effectors of apoptosis that are expressed. Although there may be a measure of mechanistic commonality between some cell types, one should always be mindful of the reality that the integrin-mediated control of cell survival and anoikis constitutes a complex business that is not only tissue type- and species context-dependent, but as well differentiation state-distinctive. Taking into account these caveats should allow for an improved focusing on the precise determinants of cell survival of a given tissue within the whole of its physiological framework, therefore facilitating an eventual elucidation of the selective integrin-mediated underpinnings that are implicated in tissue-specific apoptosis/anoikis-linked pathological disorders.
